# Dysregulation of miRNAs Targeting the IGF-1R Pathway in Pancreatic Ductal Adenocarcinoma

**DOI:** 10.3390/cells10081856

**Published:** 2021-07-22

**Authors:** Maria Dobre, Vlad Herlea, Cătălina Vlăduţ, Mihai Ciocîrlan, Vasile Daniel Balaban, Gabriel Constantinescu, Mircea Diculescu, Elena Milanesi

**Affiliations:** 1 Victor Babes National Institute of Pathology, 050096 Bucharest, Romania; maria_dobre70@yahoo.com (M.D.); elena.k.milanesi@gmail.com (E.M.); 2 Department of Pathology, Fundeni Clinical Institute, 022328 Bucharest, Romania; herlea2002@yahoo.com; 3 Department of Gastroenterology, “Prof Dr Agrippa Ionescu” Clinical Emergency Hospital, 011356 Bucharest, Romania; ciocirlan@hey.com; 4 Faculty of Medicine, Carol Davila University of Medicine and Pharmacy, 050474 Bucharest, Romania; vbalaban@yahoo.com (V.D.B.); mmdiculescu@yahoo.com (M.D.); 5 Department of Gastroenterology, Carol Davila Central Military Emergency University Hospital, 010825 Bucharest, Romania; 6 Department of Gastroenterology, Clinical Emergency Hospital Bucharest, 014461 Bucharest, Romania; gabrielconstantinescu63@gmail.com; 7 Department of Gastroenterology, Fundeni Clinical Institute, 022328 Bucharest, Romania

**Keywords:** pancreas, adenocarcinoma, microRNA, IGF-1R

## Abstract

Background: Pancreatic ductal adenocarcinoma (PDAC), the most prevalent neoplastic lethal pancreatic disease, has a poor prognosis and an increasing incidence. The insulin-like growth factor-1 receptor (IGF-1R) signaling pathway is considered to be a contributing factor to the progression, metastasis, and therapy resistance of PDAC. Currently available treatment options for PDAC are limited, but microRNAs (miRNAs) may represent a new therapeutic strategy for targeting genes involved in the IGF-1R signaling pathway. Method: We investigated the expression levels of 21 miRNAs involved in the IGF-1R signaling pathway in pancreatic tissue from 38 patients with PDAC and 11 controls (five patients with chronic pancreatitis and six patients with normal pancreatic tissue). Results: We found 19 differentially expressed miRNAs between the PDAC cases and the controls. In particular, miR-100-5p, miR-145-5p, miR-29c-3p, miR-9-5p, and miR-195-5p were exclusively downregulated in PDAC tissue but not in chronic pancreatitis or normal pancreatic tissues; both control types presented similar levels. We also identified miR-29a-3p, miR-29b-3p, and miR-7-5p as downregulated miRNAs in PDAC tissues as compared with normal tissues but not with pancreatitis tissues. Conclusions: We identified a panel of miRNAs that could represent putative therapeutic targets for the development of new miRNA-based therapies for PDAC.

## 1. Introduction

Pancreatic cancer occurs primarily as pancreatic ductal adenocarcinoma (PDAC) and represents the seventh most frequent cause of death by cancer in industrialized countries [1]. As the most common pancreatic cancer, PDAC accounts for about 85% of all cases and has a very poor prognosis, with a five-year survival rate of less than 5% [2]. This low survival rate arises largely because PDAC is usually clinically silent in its early stages; therefore, most diagnoses are made only at an advanced stage.

The pathogenesis of PDAC is not well defined; however, several risk factors have been identified, including modifiable risk factors related to lifestyle (smoking, alcohol, obesity, dietary factors, and exposure to toxic substances) and non-modifiable risk factors (sex, age, ethnicity, diabetes mellitus, family history of pancreatic cancer, genetic factors, chronic infections, and chronic pancreatitis) [3].

The histopathological progression from premalignant lesions (cystic mucinous neoplasia, intrapapillary mucinous neoplasia, pancreatic intraepithelial neoplasia, or intraductal oncocytic neoplasia) to PDAC is mediated by complex interactions that involve genetic mutations, modifications of the tumoral microenvironment, tumoral immunoediting, neoangiogenesis, and alterations in signaling pathways [4,5].

For example, the insulin and insulin-like growth factor-1 receptor (IGF-1R) signaling pathways are known to enhance the development and progression of PDAC by promoting tumor growth and metastasis and by driving therapy resistance [6]. 

The IGF-IGF-1R axis consists of three receptor tyrosine kinases: IGF-1R, IGF-2R, and insulin receptor (INSR) [7]. IGF-1R and IGF-2R, are abundant in PDAC tissue, especially in the stromal cells. Stimulation of IGF-1 activates PI-3K/Akt and Ras/Raf.MAPK cascades, and activation of the IGF-1/IGF-1R signaling pathway can have a negative impact on certain tumor suppressor proteins, such as p53, BRCA1, and VHL. IGF-1R overexpression is associated with an enhanced growth rate of cancer cells [6,8], and studies have shown that patients with advanced clinical stages of PDAC (stages II and III) have higher levels of IGF-1R and low IGFBP3, correlating with poor prognosis [9]. Although IGF-1R is critically involved in the pathophysiology of this disease, monoclonal antibody therapies targeting IGF-1R have failed to demonstrate significant clinical benefits [10]. This seems to be due both to the complexity and the homology that is shared by insulin and IGF receptors and to the knowledge gap regarding the cross-talk between cancer cells and the stroma in the pancreas [6].

Currently, available treatment options for pancreatic cancer remain limited, with most patients receiving more than one type of treatment. The primary intervention is surgical resection, followed by chemotherapy and/or radiation therapy or palliative care, depending on the stage of the cancer [11]. However, a promising new strategy for the improvement of pancreatic cancer treatment is offered by microRNAs (miRNAs), which have been identified as regulators of several cancers. Indeed, the identification of miRNA dysregulation in cancer is fundamental for identifying potential miRNA-based therapeutic targets, as well as for obtaining a better understanding of cancer pathogenesis [12]. 

Structurally, miRNAs are small single-stranded non-coding RNA molecules (18–25 nucleotides) that are involved in post-transcriptional regulation of gene expression or in inhibiting translation. It is estimated that 1–4% genes in the human genome are miRNAs [13] and control the expression of over 60% of the protein-coding genes [14]. Their role in carcinogenesis is essential, since they can promote proto-oncogene expression (acting as “oncomirs”) and/or serve a tumor suppressive function. The miRNAs can even be involved in modulating extrinsic factors, such as immune system interactions, stromal cell interactions, and even sensitivity to therapy, making these molecules the subject of intense ongoing research. 

The involvement of the IGF-1R pathway and the potential role of miRNAs in cancer therapy prompted us to seek out dysregulating miRNAs that could be involved in the insulin/IGF-1R signaling pathway, as these miRNAs may represent potential PDAC therapeutic targets. 

In this study, we evaluated tumor tissues from patients with PDAC for the expression of a panel of 21 miRNAs that target genes involved in the IGF-1R signaling pathway, and we compared the expression of these miRNAs in noncancerous tissues from patients with chronic pancreatitis or normal pancreatic tissues. The analyzed miRNAs comprised nine miRNAs belonging to the let-7 family, implicated in the control of glucose homeostasis by targeting *IGF-1R*, *IR*, and *IRS2* [15] and recently found involved in PDAC [16]. Three miRNAs regulating the expression of *IGF-1R* have been also selected (miR-100-5p [17], miR-145-5p [18], and miR-150-5p [19]) as well as miRNAs involved in pancreatic development and insulin secretion (miR-195-5p, miR-126-3p, and miR-9-5p) and miRNAs regulating diabetes-associated pancreatic cancer pathway genes (miR-19a-3p, miR-21-5p, and miR-29a/b/c-3p) [18].

## 2. Materials and Methods

### 2.1. Sample Collection

Forty-three patients with suspected pancreatic cancer underwent ultrasound endoscopy and fine needle aspiration (FNA) with a 22-gauge needle designed for standard cytologic examination. An additional aspiration was made and collected in RNA later reagent (Qiagen). The diagnosis was performed by a pathologist who identified 38 patients with PDAC and 5 patients with chronic pancreatitis (P). 

Normal pancreatic tissues were also collected from six patients who underwent surgical pancreatic resection for different reasons (neuroendocrine tumor, intraductal papillary mucinous neoplasm, adenosquamous carcinoma, and metastasis of colorectal carcinoma). The specimens were collected from normal adjacent tissue (NT) and were verified as normal by a pathologist. All samples were kept at room temperature for 24 h, and then stored at −80 °C until RNA isolation. The NT and P cases constituted our control group (CTRL). 

This study was approved by the Ethics Committee of the Clinical Emergency Hospital of Bucharest (approval number 1960 of 28 February 2019) and from the Ethics Committee of the Victor Babes National Institute of Pathology (approval number 78 of 3 December 2019). All patients signed an informed consent form before sample collection.

### 2.2. Evaluation of miRNAs

Total RNA was isolated using the miRNeasy Mini Kit (Qiagen, Hilden, Germany), according to the manufacturer’s protocol. RNA quality and quantity were assessed using a spectrophotometric method (NanoDrop 2000, Thermo Scientific, Wilmington, NC, USA). A 10 ng sample of total RNA was reverse transcribed with an miRCURY LNA RT Kit (Qiagen), and the expression of a panel including 21 miRNAs involved in the IGF-1 signaling pathway was evaluated using a miRCURY LNA SYBR Green PCR Kit and a miRCURY LNA miRNA PCR Assay (Qiagen). The 21 miRNAs were the following: hsa-let-7a-5p, hsa-let-7b-5p, hsa-let-7c-5p, hsa-let-7d-5p, hsa-miR-145-5p, hsa-miR-19a-3p, hsa-miR-195-5p, hsa-miR-126-3p, hsa-miR-150-5p, hsa-miR-29a-3p, hsa-miR-29b-3p, hsa-miR-29c-3p, hsa-miR-21-5p, hsa-miR-223-3p, hsa-miR-9-5p, hsa-miR-7-5p, hsa-let-7e-5p, hsa-let-7f-5p, hsa-let-7g-5p, hsa-let-7i-5p, and hsa-miR-100-5p. The Ct data were normalized against the geometric mean of three reference miRNAs (U6 snRNA, SNORD38B, and SNORD49A) whose stability in pancreatic normal tissue, pancreatitis, and PDAC has been validated in our samples using the RefFinder algorithm [20]. The miRNA expression data are presented as 2^−∆Ct^ values.

### 2.3. Statistical Analysis

The miRNA levels were not normally distributed (Shapiro–Wilk test <0.05); therefore, the statistical analysis was performed using nonparametric tests. The Mann–Whitney test was used to compare the miRNA expression between the PDAC and CTRL groups. The Kruskal–Wallis test, followed by pairwise comparisons, was applied to find significant differences among the PDAC, P, and NT groups. The changes in miRNA levels were considered significant if *p* < 0.05 and the fold regulation (FR) was FR > 2 or FR < −2. 

Differences in the sociodemographic features among the groups were tested with the chi-squared test (for categorical variables) and with the *t*-test (for continuous variables). Statistical analysis was performed using the Statistical Package for the Social Sciences (SPSS version 17.0). The graphs were realized using GraphPad Prism 8.4.3.

## 3. Results

This study included 49 patients, consisting of 38 patients with PDAC and 11 CTRL cases (5 P and 6 NT). The sociodemographic and lifestyle data of the patient and control groups are reported in Table 1. The two groups were homogeneous for age, sex, and lifestyle characteristics. The clinical data of the patients with PDAC are summarized in Table 2. Cholesterol and lipase level were available for 22 and 17 PDAC patients, respectively. Ten patients presented normal cholesterol levels (160.7 ± 25.4 mg/dL), five patients presented borderline levels (221.8 ± 17.2 mg/dL), and seven patients presented high levels (337.7 ± 91.3 mg/dL). An impairment of lipase level was observed in six patients, reporting a lipase average of 902.6 ± 697.4 U/L.

**Figure 1:**
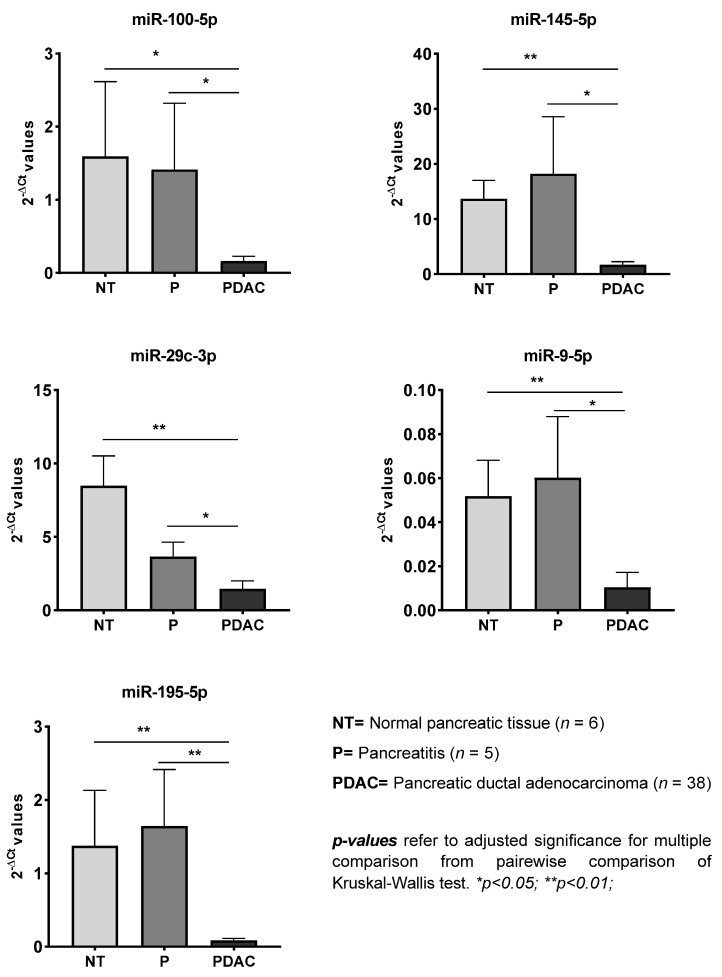
Differential expression of miRNAs in pancreatic tissues from normal (NT), chronic pancreatitis (P), and pancreatic ductal adenocarcinoma (PDAC) groups. Values are shown as mean ± SEM.

First, we compared the PDAC and control groups for the levels of the 21 selected miRNAs involved in the IGF-1R pathway. The PDAC group showed downregulation of 19 of these 21 miRNAs (*p* < 0.05, FR < −2.00) (Supplementary data). Further analysis of the three groups showed that miR-100-5p, miR-145-5p, miR-29c-3p, miR-9-5p, and miR-195-5p were significantly downregulated in PDAC patients as compared with the NT and P groups (Figure 1).

**Figure 2:**
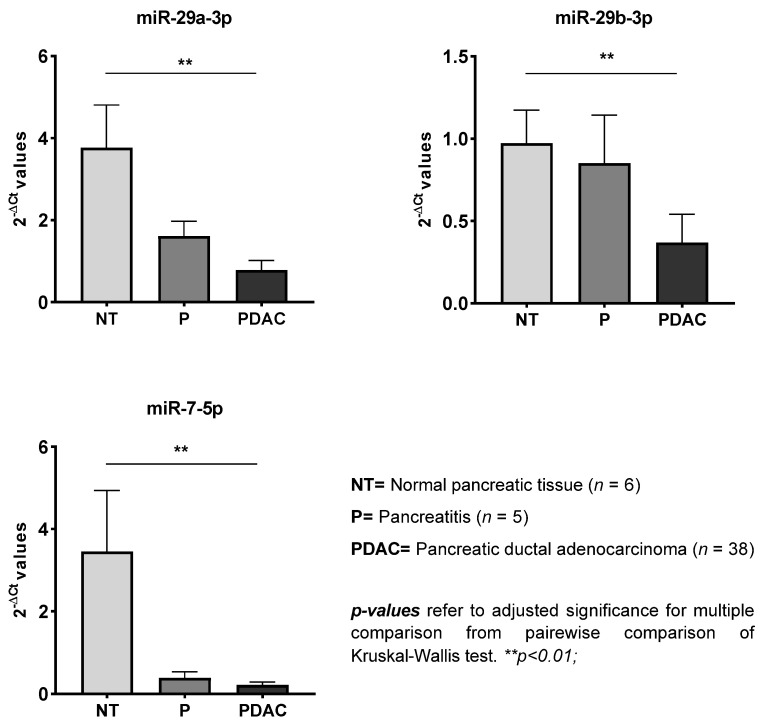
Differential expression of miRNAs in pancreatic tissues from normal (NT) and pancreatic ductal adenocarcinoma (PDAC) groups. Values are shown as mean ± SEM.

Three miRNAs (miR-29a-3p, miR29b-3p, and miR-7-5p) were differentially expressed in the PDAC group as compared with the NT group, but no significant changes were found in the P group (Figure 2). 

The differences in miR-let-7a-5p, miR-let-7b-5p, miR-let-7c-5p, miR-let-7d-5p, miR-let-7g-5p, miR-let-7f-5p, miR-126-3p, miR-150-5p, miR-19a-3p, miR-21-5p, and miR-let-7e-5p expression were significant only in the comparison between the PDAC and P groups (Supplementary Figure S1). No difference was observed in the expression of the 19 miRNAs between the P and NT groups (Table 3).

At the moment of miRNAs analysis, nine PDAC patients were still alive and data regarding the survival were available for the other 29 individuals. The survival range was between 1 and 12 months and the average (±SD) was 5.2 ± 3.3 months. Sixteen patients reported a survival below 6 months and 13 a survival above/equal to 6 months. No significant correlations were found between the months of survival and the analyzed miRNAs level. Moreover, the level of these miRNAs did not change (*p* > 0.05) between the group of survival <6 months (*n* = 16) and survival ≥6 months (*n* = 13). Additionally, no significant differences in miRNA expression were found in the PDAC group in relation to tumor localization, clinical stage, lymph node invasion, or the presence of diabetes or jaundice (data not shown).

## 4. Discussion

Pancreatic cancer remains one of the most aggressive malignancies and shows poor prognosis and a disappointing response to therapeutic drugs. Previous studies have identified an association between the progression of PDAC and the overexpression of several growth factor receptors, such as HER [21], EGFR [22], and IGF-1R [23,24,25]. IGF-1R overexpression has been correlated with poor prognosis [9], high tumor grade, and short overall survival [26]. In the last decade, several IGF/IGF-1R inhibitors, including monoclonal antibodies against IGF-1R and against its ligands (IGF-1 and IGF-2) and inhibitors of the IGF-1R tyrosine kinase, have been introduced in clinical trials, but these have failed to show clinical benefit in the patient population [27]. However, IGF-1R remains a valid target, and new treatment strategies targeting its pathway need further development. 

In this context, the targeting of miRNAs, which are able to regulate multiple genes, may achieve a cumulative effect on a set of related target proteins at multiple levels of the same pathway. The miRNAs, therefore, represent an attractive therapeutic target [28], and several candidates were identified in the present study by the analysis of the panel of 21 miRNAs targeting genes involved in the IGF-1R signaling pathway. In particular, miR-100-5p, miR-145-5p, miR-29c-3p, miR-9-5p, and miR-195-5p were exclusively downregulated in PDAC tissue but not in the chronic pancreatitis and normal pancreatic tissues. 

Downregulation of miR-100-5p has been reported in different types of cancers, including oral cancer cells, where it contributes to malignancy [29] and in the serum of patients with bladder cancer [30] and prostate cancer [31]. This miRNA also seems to regulate the mechanism of apoptosis in breast cancer [32], and the levels of miR-100 were significantly lower in the serum/plasma or peripheral blood mononuclear cells from patients with type I diabetes [33] and in serum from obese normoglycemic subjects and subjects with type II diabetes as compared with controls [34]. Interestingly, in line with our finding of downregulation of miR-100-5p in PDAC tissue, other studies have suggested that this miRNA inhibits the proliferation and invasion of PDAC [35].

A few previous studies have investigated a role for miR-145-5p in PDAC. For example, in 2014, miR-145 was suggested to act as a tumor suppressor and regulator of MUC13, which is aberrantly overexpressed in pancreatic cancer [36]. Two years later, an association was found for a panel of 19 miRNAs, including miR-145-star, and the overall survival and disease-free survival in 104 patients with pancreatic tumors [37]. In support of these results, and in line with our findings, a recent study in an animal model demonstrated that the overexpression of miR-145-5p reduced the in vivo growth of xenograft PDAC tumors [38]. 

The miR-29 family members, which comprise miR-29a, miR-29b (1 and 2), and miR 29c, have all been linked to pancreatic cancer [39]. For example, miR-29a showed a tumor-suppressive role that involved targeting of MUC1, an oncogenic mucin that is overexpressed in PDAC and other epithelial cancers [40]. This miRNA functions as a potent autophagy inhibitor in PDAC and increases the effect of gemcitabine in cancer cells, while decreasing cancer cell invasion [41]. Similarly, miR-29b also appears to act as a tumor suppressor that targets DNA methyltransferase 3b to decrease cell viability and promote apoptosis of pancreatic cancer [42]. Notably, miR-29b also targets *IGF-1* and the PI3K signaling pathway (p85α) [43]. Likewise, miR-29c suppresses cell migration and invasion by targeting *MMP2*, and this miRNA has been suggested as a novel marker of pancreatic cancer metastasis [44]. Interestingly, all three miR-29 family members were downregulated in the PDAC cases in our study, but only miR-29c was differentially expressed in the PDAC tissues as compared with the chronic pancreatitis and normal tissues. Differential expression of miR-29a and miR29b was observed for PDAC as compared with the normal tissues, but not when compared to the pancreatitis tissues. Moreover, it has been shown that miR-29 is a key regulator of collagen expression [45] and the loss of miR-29 is a common phenomenon of activated pancreatic stellate cells (PSCs)/fibroblasts, the major stromal cells responsible for fibrotic stromal reaction [46]. The literature data regarding the involvement of miR-9-5p in PDAC are scarce, but this miRNA is involved in insulin secretion [47] and may represent a prognostic or therapeutic target in pancreatic cancer. Its low expression has been strongly correlated with poor overall survival, and its overexpression markedly inhibited pancreatic cancer cell proliferation by enhancing cell apoptosis [48].

Studies on miR-195-5p have demonstrated that this miRNA suppresses the expression of IGF1R and the activation of PI3K/Akt signaling [49], but its role in PDAC has not been extensively investigated. One study suggested that the overexpression of miR-195 inhibits the proliferation, migration, and invasion of pancreatic cancer cells and that its levels inversely correlate with doublecortin-like kinase 1, a tumor-specific stem cell marker in pancreatic cancer [50]. A few recent studies have reported that specific long noncoding RNAs drive tumorigenesis by modulating the miR-195-5p/Wnt/beta-catenin signaling pathway [51] and promote the progression of pancreatic cancer by upregulating programmed death-ligand 1 by acting as a sponge for microRNA-195-5p [52]. Higher levels of miR-195 have also recently been reported in extracellular vesicles from blood from patients with PDAC than from blood from healthy controls, although no difference was detected when compared to extracellular vesicles from blood from patients with chronic pancreatitis [53].

In conclusion, our findings validated previous results that indicated the involvement of a panel of miRNAs targeting genes involved in the IGF-1R signaling pathway. Specifically, we showed the downregulation of miR-100-5p, miR-145-5p, miR-29c-3p, miR-9-5p, and miR-195-5p exclusively in PDAC tissue as compared with chronic pancreatitis and normal pancreatic tissues. These miRNAs could, therefore, be considered potential molecular targets for promising miRNA-based approaches for future curative treatments for PDAC.

All patients in the present study were followed up, including the control group with chronic pancreatitis, since in these particular cases FNA offers less information that fine needle biopsy or surgery. The study was limited by its relatively small cohort; therefore, further investigations on larger numbers of patients are needed to confirm the results presented here.

## Figures and Tables

**Table 1:** Sociodemographic and lifestyle data of patients and controls enrolled in the study.

Features	PDAC (*n* = 38)	CTRL (*n* = 5 P, *n* = 6 NT)	Significance
Age (mean ± SD)	65.08 ± 9.93	CTRL = 58.00 ± 13.93	PDAC vs. CTRL, *p* = 0.064
		P = 65.80 ± 13.36	PDAC vs. P, *p* = 0.884
		NT = 51.50 ± 11.55	PDAC vs. NT, *p =* 0.004
Sex (%F)	55.30%	CTRL = 36.4%	PDAC vs. CTRL, χ = 1.219, *p* = 0.269
		P = 40%	PDAC vs. P, χ = 0.414, *p* = 0.520
		NT = 33.3%	PDAC vs. NT, χ = 0.999, *p* = 0.318
Smokers (%)	39.50%	CTRL = 27.3%	PDAC vs. CTRL, χ = 0.546, *p* = 0.460
		P = 0%	PDAC vs. P, χ = 3.031, *p* = 0.082
		NT = 50%	PDAC vs. NT, χ = 0.238, *p* = 0.626
Coffee consumers (%)	39.50%	CTRL = 63.7%	PDAC vs. CTRL, χ = 2.013, *p* = 0.156
		P = 60%	PDAC vs. P, χ = 0.765, *p* = 0.382
		NT = 66.7%	PDAC vs. NT, χ = 1.562, *p* = 0.211
Alcohol consumers (%)	27.30%	CTRL = 15.8 %	PDAC vs. CTRL, χ = 0.750, *p* = 0.386
		P = 40%	PDAC vs. P, χ = 1.710, *p* = 0.191
		NT = 16.7%	PDAC vs. NT, χ = 0.003, *p* = 0.956

**Table 2:** Clinical features of PDAC patients.

PDAC (*n* = 38)						
Localization	Clinical Stage	Lymph Node Invasion	Metastasis	Diabetes	Jaundice	Family History of PDAC
Head = 23	T2 = 7	18	14	10	15	2
	T3 = 6					
	T4 = 10					
Neck = 3	T2 = 1	2	1	2	0	0
	T3 = 2					
Body = 6	T2 = 2	5	5	3	0	0
	T3 = 2					
	T4 = 2					
Body-Tail = 4	T2 = 2	2	2	2	0	1
	T3 = 1					
	T4 = 1					
Tail = 2	T3 = 1	2	2	0	0	0
	T4 = 1					

**Table 3:** Comparison of the levels of the miRNAs in PDAC, NT, and P. *p*-Values refer to Kruskal–Wallis test followed by pairwise comparisons. FR (fold regulation); ns (statistically not significant).

miRNA	Significance	PDAC vs. NT		PDAC vs. P		P vs. NT	
	*p*-Value	FR	*p*-Value	FR	*p*-Value	FR	*p*-Value
miR-let-7a-5p	0.006	/	ns	−19.01	0.005	*ns*	*ns*
miR-let-7b-5p	0.002	/	ns	−19.70	0.006	*ns*	*ns*
miR-let-7c-5p	0.004	/	ns	−12.58	0.005	*ns*	*ns*
miR-let-7d-5p	0.012	/	ns	−13.48	0.009	*ns*	*ns*
miR-let-7g-5p	0.034	/	ns	−12.64	0.044	*ns*	*ns*
miR-100-5p	0.001	−9.83	0.012	−8.72	0.035	*ns*	*ns*
miR-let-7f-5p	0.015	/	ns	−12.44	0.015	*ns*	*ns*
miR-126-3p	0.002	/	ns	−29.16	0.007	*ns*	*ns*
miR-145-5p	<0.001	−8.15	0.003	−10.84	0.019	*ns*	*ns*
miR-150-5p	0.002	/	ns	−23.70	0.002	*ns*	*ns*
miR-19a-3p	0.007	/	ns	−10.13	0.011	*ns*	*ns*
miR-21-5p	0.012	/	ns	−2.96	0.025	*ns*	*ns*
miR-29a-3p	0.001	−4.82	0.003	/	ns	*ns*	*ns*
miR-29b-3p	0.001	−2.64	0.006	/	ns	*ns*	*ns*
miR-29c-3p	<0.001	−5.78	0.001	−2.49	0.04	*ns*	*ns*
miR-7-5p	0.001	−16.41	0.001	/	ns	*ns*	*ns*
miR-9-5p	<0.001	−4.96	0.002	−5.76	0.013	*ns*	*ns*
miR-let-7e-5p	0.006	/	ns	−5.52	0.015	*ns*	*ns*
miR-195-5p	<0.001	−16.09	0.001	−19.26	0.004	*ns*	*ns*

ns: not significant.

## Data Availability

Data is contained within this article and the Supplementary Materials. Raw data can be obtained from the corresponding author upon reasonable request.

## Supplementary Materials

The following are available online at https://www.mdpi.com/article/10.3390/cells10081856/s1, Figure S1: Differential expression of miRNAs in pancreatic tissues from chronic pancreatitis (P), and pancreatic ductal adenocarcinoma (PDAC) groups. Values are shown as mean ± SEM.

## References

[1] Bray F., Ferlay J., Soerjomataram I., Siegel R.L., Torre L.A., Jemal A. (2018). Global cancer statistics 2018: GLOBOCAN estimates of incidence and mortality worldwide for 36 cancers in 185 countries. CA Cancer J. Clin..

[2] Foucher E.D., Ghigo C., Chouaib S., Galon J., Iovanna J., Olive D. (2018). Pancreatic Ductal Adenocarcinoma: A strong imbalance of good and bad immunological cops in the tumor microenvironment. Front. Immunol..

[3] Rawla P., Sunkara T., Gaduputi V. (2019). Epidemiology of pancreatic cancer: Global trends, etiology and risk factors. World J. Oncol..

[4] Fanta P.T., Lowy A.M. (2015). Adenocarcinoma of the Pancreas. Yamada’ s Textbook of Gastroenterology.

[5] Kim J.Y., Hong S.-M. (2018). Precursor lesions of pancreatic cancer. Oncol. Res. Treat..

[6] Mutgan A.C., Besikcioglu H.E., Wang S., Friess H., Ceyhan G.O., Demir I.E. (2018). Insulin/IGF-driven cancer cell-stroma crosstalk as a novel therapeutic target in pancreatic cancer. Mol. Cancer.

[7] Yuan J., Yin Z., Tao K., Wang G., Gao J. (2018). Function of insulin-like growth factor 1 receptor in cancer resistance to chemotherapy. Oncol. Lett..

[8] Kopantzev E.P., Kopantseva M.R., Grankina E.V., Mikaelyan A., Egorov V.I., Sverdlov E.D. (2019). Activation of IGF/IGF-IR signaling pathway fails to induce epithelial-mesenchymal transition in pancreatic cancer cells. Pancreatology.

[9] Hirakawa T., Yashiro M., Murata A., Hirata K., Kimura K., Amano R., Yamada N., Nakata B., Hirakawa K. (2013). IGF-1 receptor and IGF binding protein-3 might predict prognosis of patients with resectable pancreatic cancer. BMC Cancer.

[10] Camblin A.J., Pace E.A., Adams S., Curley M.D., Rimkunas V., Nie L., Tan G., Bloom T., Iadevaia S., Baum J. (2018). Dual Inhibition of IGF-1R and ErbB3 Enhances the Activity of Gemcitabine and Nab-Paclitaxel in Preclinical Models of Pancreatic Cancer. Clin. Cancer Res..

[11] Pereira N.P., Corrêa J.R. (2018). Pancreatic cancer: Treatment approaches and trends. J. Cancer Metastasis Treat..

[12] Słotwiński R., Lech G., Słotwińska S.M. (2018). MicroRNAs in pancreatic cancer diagnosis and therapy. Cent. J. Immunol..

[13] Esquela-Kerscher A., Slack F.J. (2006). Oncomirs—MicroRNAs with a role in cancer. Nat. Rev. Cancer.

[14] Catalanotto C., Cogoni C., Zardo G. (2016). MicroRNA in control of gene expression: An overview of Nuclear Functions. Int. J. Mol. Sci..

[15] Chen B., Li J., Chi D., Sahnoune I., Calin S., Girnita L., Calin G.A. (2019). Non-Coding RNAs in IGF-1R signaling regulation: The underlying pathophysiological link between diabetes and cancer. Cells.

[16] Nweke E.E., Brand M. (2020). Downregulation of the let-7 family of microRNAs may promote insulin receptor/insulin-like growth factor signalling pathways in pancreatic ductal adenocarcinoma. Oncol. Lett..

[17] Huang J.S., Egger M.E., Grizzle W.E., McNally L.R. (2013). MicroRNA-100 regulates IGF1-receptor expression in metastatic pancreatic cancer cells. Biotech. Histochem..

[18] Chakraborty C., George Priya Doss C., Bandyopadhyay S. (2013). miRNAs in insulin resistance and diabetes-associated pancreatic cancer: The “minute and miracle” molecule moving as a monitor in the “genomic galaxy”. Curr. Drug Targets.

[19] Farhana L., Dawson M.I., Murshed F., Das J.K., Rishi A.K., Fontana J.A. (2013). Upregulation of miR-150* and miR-630 induces apoptosis in pancreatic cancer cells by targeting IGF-1R. PLoS ONE.

[20] Xie F., Xiao P., Chen D., Xu L., Zhang B. (2012). miRDeepFinder: A miRNA analysis tool for deep sequencing of plant small RNAs. Plant Mol. Biol..

[21] Hirakawa T., Nakata B., Amano R., Kimura K., Shimizu S., Ohira G., Yamada N., Ohira M., Hirakawa K. (2011). HER3 overexpression as an independent indicator of poor prognosis for patients with curatively resected pancreatic cancer. Oncology.

[22] Komoto M., Nakata B., Nishii T., Kawajiri H., Shinto O., Amano R., Yamada N., Yashiro M., Hirakawa K. (2010). In vitro and in vivo evidence that a combination of lapatinib plus S-1 is a promising treatment for pancreatic cancer. Cancer Sci..

[23] Nair P.N., De Armond D.T., Adamo M.L., Strodel W.E., Freeman J.W. (2001). Aberrant expression and activation of insulin-like growth factor-1 receptor (IGF-1R) are mediated by an induction of IGF-1R promoter activity and stabilization of IGF-1R mRNA and contributes to growth factor independence and increased survival of the panc. Oncogene.

[24] Lin Y., Rong L., Zhao J., Lin R., Li S. (2018). MicroRNA-539 inhibits cell proliferation, colony formation and invasion in pancreatic ductal adenocarcinoma by directly targeting IGF-1R. Mol. Med. Rep..

[25] Du C., da Silva A., Morales-Oyarvide V., Dias Costa A., Kozak M.M., Dunne R.F., Rubinson D.A., Perez K., Masugi Y., Hamada T. (2020). Insulin-like growth factor-1 receptor expression and disease recurrence and survival in patients with resected pancreatic ductal adenocarcinoma. Cancer Epidemiol. Biomark. Prev..

[26] Valsecchi M.E., McDonald M., Brody J.R., Hyslop T., Freydin B., Yeo C.J., Solomides C., Peiper S.C., Witkiewicz A.K. (2012). Epidermal growth factor receptor and insulinlike growth factor 1 receptor expression predict poor survival in pancreatic ductal adenocarcinoma. Cancer.

[27] Chen H.X., Sharon E. (2013). IGF-1R as an anti-cancer target--trials and tribulations. Chin. J. Cancer.

[28] Shah M.Y., Calin G.A. (2014). MicroRNAs as therapeutic targets in human cancers. Wiley Interdiscip. Rev. RNA.

[29] Henson B.J., Bhattacharjee S., O’Dee D.M., Feingold E., Gollin S.M. (2009). Decreased expression of miR-125b and miR-100 in oral cancer cells contributes to malignancy. Genes. Chromosomes Cancer.

[30] Motawi T.K., Rizk S.M., Ibrahim T.M., Ibrahim I.A.-R. (2016). Circulating microRNAs, miR-92a, miR-100 and miR-143, as non-invasive biomarkers for bladder cancer diagnosis. Cell Biochem. Funct..

[31] Wang M., Ren D., Guo W., Wang Z., Huang S., Du H., Song L., Peng X. (2014). Loss of miR-100 enhances migration, invasion, epithelial-mesenchymal transition and stemness properties in prostate cancer cells through targeting Argonaute 2. Int. J. Oncol..

[32] Gong Y., He T., Yang L., Yang G., Chen Y., Zhang X. (2015). The role of miR-100 in regulating apoptosis of breast cancer cells. Sci. Rep..

[33] Assmann T.S., Recamonde-Mendoza M., De Souza B.M., Crispim D. (2017). MicroRNA expression profiles and type 1 diabetes mellitus: Systematic review and bioinformatic analysis. Endocr. Connect..

[34] Pek S.L.T., Sum C.F., Lin M.X., Cheng A.K.S., Wong M.T.K., Lim S.C., Tavintharan S. (2016). Circulating and visceral adipose miR-100 is down-regulated in patients with obesity and Type 2 diabetes. Mol. Cell. Endocrinol..

[35] Gong R., Jiang Y. (2020). Non-coding RNAs in Pancreatic Ductal Adenocarcinoma. Front. Oncol..

[36] Khan S., Ebeling M.C., Zaman M.S., Sikander M., Yallapu M.M., Chauhan N., Yacoubian A.M., Behrman S.W., Zafar N., Kumar D. (2014). MicroRNA-145 targets MUC13 and suppresses growth and invasion of pancreatic cancer. Oncotarget.

[37] Namkung J., Kwon W., Choi Y., Yi S.G., Han S., Kang M.J., Kim S.-W., Park T., Jang J.-Y. (2016). Molecular subtypes of pancreatic cancer based on miRNA expression profiles have independent prognostic value. J. Gastroenterol. Hepatol..

[38] Ding Y., Cao F., Sun H., Wang Y., Liu S., Wu Y., Cui Q., Mei W., Li F. (2019). Exosomes derived from human umbilical cord mesenchymal stromal cells deliver exogenous miR-145-5p to inhibit pancreatic ductal adenocarcinoma progression. Cancer Lett..

[39] Alizadeh M., Safarzadeh A., Beyranvand F., Ahmadpour F., Hajiasgharzadeh K., Baghbanzadeh A., Baradaran B. (2019). The potential role of miR-29 in health and cancer diagnosis, prognosis, and therapy. J. Cell. Physiol..

[40] Tréhoux S., Lahdaoui F., Delpu Y., Renaud F., Leteurtre E., Torrisani J., Jonckheere N., Van Seuningen I. (2015). Micro-RNAs miR-29a and miR-330-5p function as tumor suppressors by targeting the MUC1 mucin in pancreatic cancer cells. Biochim. Biophys. Acta.

[41] Kwon J.J., Willy J.A., Quirin K.A., Wek R.C., Korc M., Yin X.-M., Kota J. (2016). Novel role of miR-29a in pancreatic cancer autophagy and its therapeutic potential. Oncotarget.

[42] Wang L.-H., Huang J., Wu C.-R., Huang L.-Y., Cui J., Xing Z.-Z., Zhao C.-Y. (2018). Downregulation of miR-29b targets DNMT3b to suppress cellular apoptosis and enhance proliferation in pancreatic cancer. Mol. Med. Rep..

[43] Li J., Chan M.C., Yu Y., Bei Y., Chen P., Zhou Q., Cheng L., Chen L., Ziegler O., Rowe G.C. (2017). miR-29b contributes to multiple types of muscle atrophy. Nat. Commun..

[44] Zou Y., Li J., Chen Z., Li X., Zheng S., Yi D., Zhong A., Chen J. (2015). miR-29c suppresses pancreatic cancer liver metastasis in an orthotopic implantation model in nude mice and affects survival in pancreatic cancer patients. Carcinogenesis.

[45] Maurer B., Stanczyk J., Jüngel A., Akhmetshina A., Trenkmann M., Brock M., Kowal-Bielecka O., Gay R.E., Michel B.A., Distler J.H.W. (2010). MicroRNA-29, a key regulator of collagen expression in systemic sclerosis. Arthritis Rheum..

[46] Kwon J.J., Nabinger S.C., Vega Z., Sahu S.S., Alluri R.K., Abdul-Sater Z., Yu Z., Gore J., Nalepa G., Saxena R. (2015). Pathophysiological role of microRNA-29 in pancreatic cancer stroma. Sci. Rep..

[47] Ma J., Wu Y., He Y. (2021). Silencing circRNA LRP6 down-regulates PRMT1 to improve the streptozocin-induced pancreatic β-cell injury and insulin secretion by sponging miR-9-5p. J. Bioenerg. Biomembr..

[48] Wang J., Wang B., Ren H., Chen W. (2019). miR-9-5p inhibits pancreatic cancer cell proliferation, invasion and glutamine metabolism by targeting GOT1. Biochem. Biophys. Res. Commun..

[49] Du P., Liu F., Liu Y., Shao M., Li X., Qin G. (2020). Linc00210 enhances the malignancy of thyroid cancer cells by modulating miR-195-5p/IGF1R/Akt axis. J. Cell. Physiol..

[50] Zhou B., Sun C., Hu X., Zhan H., Zou H., Feng Y., Qiu F., Zhang S., Wu L., Zhang B. (2017). MicroRNA-195 suppresses the progression of pancreatic cancer by targeting DCLK1. Cell. Physiol. Biochem..

[51] Wu X., Xia T., Cao M., Zhang P., Shi G., Chen L., Zhang J., Yin J., Wu P., Cai B. (2019). LncRNA BANCR promotes pancreatic cancer tumorigenesis via modulating MiR-195-5p/Wnt/β-catenin signaling pathway. Technol. Cancer Res. Treat..

[52] Zhou W.-Y., Zhang M.-M., Liu C., Kang Y., Wang J.-O., Yang X.-H. (2019). Long noncoding RNA LINC00473 drives the progression of pancreatic cancer via upregulating programmed death-ligand 1 by sponging microRNA-195-5p. J. Cell. Physiol..

[53] Zeöld A., Sándor G.O., Kiss A., Soós A.Á., Tölgyes T., Bursics A., Szűcs Á., Harsányi L., Kittel Á., Gézsi A. (2021). Shared extracellular vesicle miRNA profiles of matched ductal pancreatic adenocarcinoma organoids and blood plasma samples show the power of organoid technology. Cell. Mol. Life Sci..

