# Supplementary material for: Dysregulation of miRNAs Targeting the IGF-1R Pathway in Pancreatic Ductal Adenocarcinoma
Source: Cells. 2021 Jul 22;10(8):1856. doi: 10.3390/cells10081856 (PMC8391367; doi:10.3390/cells10081856)
Supplement: Supplementary file 1 [file cells-10-01856-s001.zip › cells-1300694-supplementary.pdf]

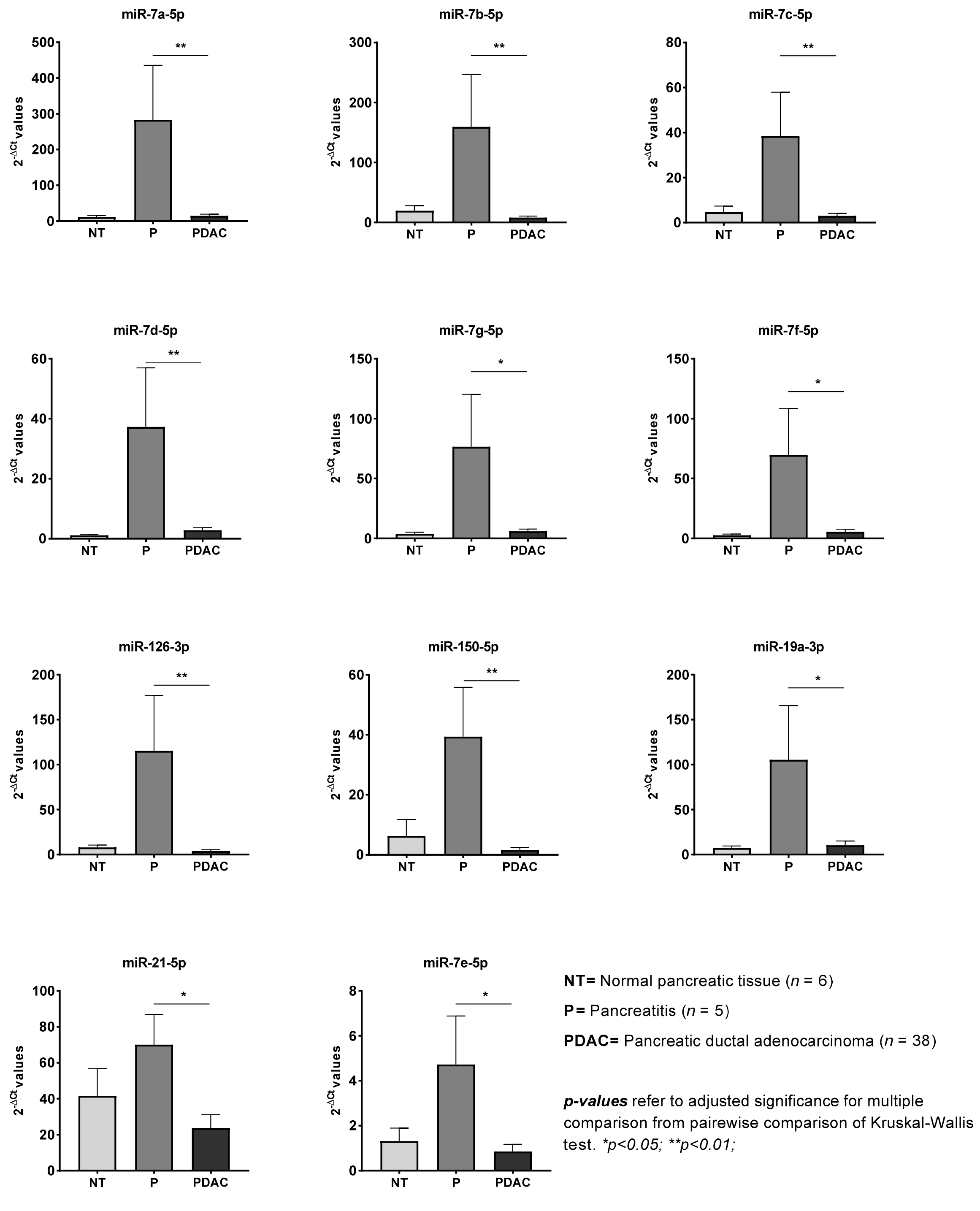

Supplementary Figure 1: Differential expression of miRNAs in pancreatic tissues from chronic pancreatitis (P), and pancreatic ductal adenocarcinoma (PDAC) groups. Values are shown as mean ± SEM.
